# Harmonizing interactions between clinical trial sponsors and study research sites: An appraisal of the key challenges and recommendations for addressing them

**DOI:** 10.1017/cts.2026.10734

**Published:** 2026-04-01

**Authors:** Kesley Holmes, Kasey Boynton, Monique Adams, Jenny Garcia, Priscilla Pemu

**Affiliations:** 1 Clinical Research Center, Morehouse School of Medicine, Atlanta, GA, USA; 2 Sanofi US Services Inc., Research & Deveolpment, Bridgewater, NJ, USA; 3 ICON PLC, Blue Bell, PA, USA

**Keywords:** Administration, contract research organization, trial management, site-sponsor collaboration, workforce

## Abstract

Successful completion of industry-sponsored clinical trials requires effective collaboration between sponsors and clinical research sites recruiting patients. As pharmaceutical companies specialize in more therapy areas, complexity and volume of clinical trials increases, with study sites facing growing operational and logistical challenges. These may be administrative, financial, technological, or workforce-related and can prevent sites from meeting trial obligations, inhibiting long-term site sustainability. Here we outline a suggested framework (with metrics) designed to address three key pillars: site infrastructure, workforce, and the establishment of a ‘trial funnel’ to maintain sufficient trial volume. We review key site-level challenges and barriers to success in clinical trial conduct and argue that issues could be mitigated by sponsors investing programmatically in their site partnerships, including investing in research-naïve sites. Long-term programmatic planning and investment has the potential to deliver greater efficiency and sustainability in trial delivery; site investment upfront would increase working capital for the site, maximizing commitment and security on both sides. This, however, requires safeguarding through the implementation of targets and metrics of success. Many of the challenges faced in modern clinical research can be mitigated by new and longer-term thinking, concerning the working relationship and methods adopted between sponsors and research sites.

## Introduction

Clinical trials are gold standard and required for drug approvals. The number of clinical trials conducted has increased consistently over time, and by over 30% since 2020 [1]. To keep up with this growth, there is a need for new site development. Since most clinical trials are conducted at large research centers, sponsors should be partners in helping more research-naïve sites become sustainable research centers.

Pharmaceutical companies depend on clinical investigators and their site staff to identify and enroll eligible patients for their trials. However, there are often inefficiencies in these interactions that create operational burdens for sites and affect their ability to sustain ongoing participation in trials. One reason is that the primary role (and revenue source) of investigative sites is patient care rather than conducting trials [2]. As a result, processes become increasingly complex, and clinical trials can pose sizeable burdens on research sites [3]. It is therefore important to recognize the key challenges that sites face so that strategies can be implemented to mitigate them.

Although engaging principal investigators (PIs) is a key step for sponsors to instigate trials, the long-term sustainability of a clinical trial site depends equally on robust operational management and ensuring the regulatory compliance of procedures [4]. Many sponsors support sites with ethical considerations of trial conduct, including standard operating procedures (SOPs), adherence to the requirements of the International Council for Harmonisation of Technical Requirements of Pharmaceuticals for Human Use (ICH), Good Clinical Practice (GCP), protocol adherence, and patient recruitment. Despite this, however, there may be limited operational resources available to help sites to develop the administrative skills required to become sustainable research centers.

To optimize site engagement and operational support, greater collaboration with site administrators and leadership may be necessary. Moving beyond the current trial-by-trial approach, a sustainable, long-term partnership model between sponsors, contract research organizations (CROs), and research sites could enhance trust, transparency, and trial success [5–7]. In addition, clinical trial sites should be managed as a business [8], with strategy and scalability in mind for the best chance at long-term sustainability.

In this article we aim to review insights from surveys of clinical research sites by the WIRB-Copernicus Group (WCG) [3] and the Society for Clinical Research Sites (SCRS) [9,10] of the key challenges and operational barriers impacting them. We review and build upon recommendations from the SCRS for ways in which sponsors/CROs can reduce the operational barriers that sites currently face and how a successful partnership can support the long-term viability of individual sites.

## Key operational barriers to clinical research site sustainability and proposed recommendations

Operational barriers to clinical trial conduct at study sites can be administrative, financial, or due to challenges with the workforce or technological demands [3,10,11]. The existence of these barriers greatly narrows the potential patient funnel for recruitment, which in turn results in delayed drug development, reduced patient access to trials, and increased costs [3].

**Figure 1.**
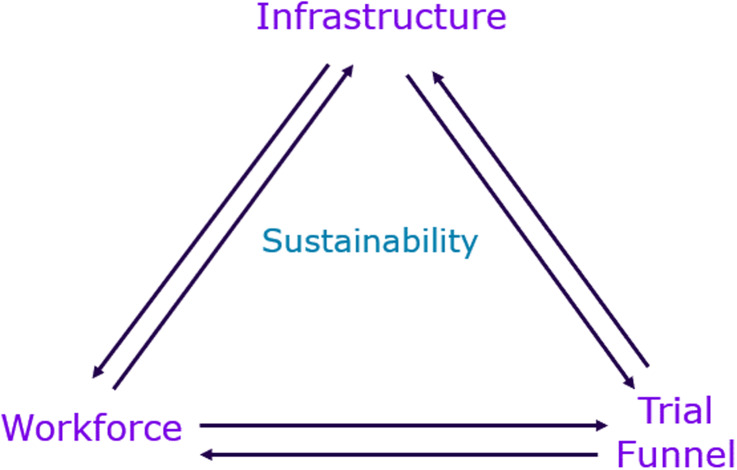
Framework for measures/predictors of clinical research site success.

Here, we identify these key operational barriers and provide recommendations for ways in which sponsors can support their study sites to build site capacity and investment to enable long-term partnerships and clinical trial activity. To support optimal implementation of these recommendations, we provide a framework divided into three key pillars–infrastructure, workforce, and trial funnel (Figure 1), with metrics to measure and track the performance of the sponsor-site partnership. While the framework is presented primarily from the sponsor perspective, it is intentionally designed to be bidirectional. In addition to supporting sponsors in assessing site capabilities and partnership readiness, the framework also provides clinical research sites with insight into sponsor expectations and identifies operational indicators that may guide site development and long-term sustainability. Considerations related to quality oversight, regulatory compliance, and a culture of quality underpin all pillars of the framework and are treated as foundational rather than as standalone metrics.

The metrics are intentionally high-level and non-prescriptive, allowing flexibility in how they are operationalized by sponsors and site leadership teams across different organizational contexts, site maturity levels, and trial complexities. The proposed metrics are intended to support both an initial assessment of site readiness and, where appropriate, ongoing evaluation over time. While presented from a sponsor perspective, the framework is also designed to be used collaboratively, enabling clinical research sites to self-assess capacity and align development efforts with sponsor expectations that support decision-making.

To illustrate how the proposed metrics may be applied in practice, workforce stability may be assessed using staff retention or turnover rates at a study site. Such information can be used to identify resourcing risks, inform discussions between sponsors and sites regarding support needs, and guide decisions related to targeted investment or capacity-building efforts over time. This example is intended to demonstrate practical application of the framework rather than prescribe a specific measurement approach.

In the case of infrastructure, it is critical that investigators engage with administrative leadership. Site success should be dependent on efficient operational processes and technology, with metrics relating to facilities, equipment, processes, training plans, technology, and the financial health required to support trial activity (Table 1).

**Table 1.** Measures/predictors of site success: infrastructure

	Metric(s) for success	Goal(s), site has:
Facilities	Space for research and storage	Modern pharmacy Patient-friendly space Document storage
Equipment	Equipment to support advanced clinical capabilities	Appropriate equipment certifications (CLIA, etc.) Investment in digitized equipment Clinical capabilities (iPads, laptops, thermometers, etc.) Centrifuge Regular process improvement and upgrade plan in place Specimen and materials management process exist
Processes	SOPs related to the management of industry-sponsored clinical studies	Processes documented, measured, and controlled Continuous improvement process in place Understands “how to get things done” at respective centers
Training plans	Internal comprehensive training programs for research and staff	ICH/GCP and diversity training Operational training plans for staff onboarding Investigator training strategy and plan
Technology	Investment in digital and technology capabilities	Clinical trial management system Access to electronic medical records integrated with total healthcare system Pre-screening tool Telehealth and virtual visit options Use of digital communication tools Patients track and manage their care online Digital tools for scheduling appointments
Financial health	Financial runway	Financial viability to support research and administrative staff outside of study budgets >6 months Working capital

CLIA = Clinical Laboratory Improvement Amendments; GCP = Good Clinical Practice; ICH = International Council for Harmonisation of Technical Requirements of Pharmaceuticals for Human Use; SOP = standard operating procedure.

Regarding workforce, a central team concerned with clinical research conduct should incentivize all those involved and provide a transparent career growth ladder. Metrics for success should relate to administrative leadership engagement, operational mentorship, and investigator engagement. In addition, the metrics should assess the presence of models for sourcing and training junior investigators, succession planning, and depth of experience, and for ensuring that resource and staff diversity are aligned to trial requirements (Table 2).

**Table 2.** Measures/predictors of site success: workforce

	Metrics for success	Goal(s), site has
Administrative leadership engagement	Strong organizational alignment on the value in engaging in clinical research	Metrics for achievement on research capacity and goals Plans for growth and expansion of research capacity
Operational mentorship	Mentors outside the site with experience managing profitable research centers	Regular mentoring partnership/collaboration with established operational leaders from other sites and centers Professional development opportunities for operational leaders through organizations like SCRS and ACRP
Investigator engagement	Interest from PIs to conduct research	PI-specific performance targets and goals for engaging in industry-sponsored research
Junior investigators	Model to source and train junior investigators	At least one junior investigator staffed Formal training program for upskilling junior investigators
Resource model	Resource model aligned to trial complexity	Mature resourcing model in addition to office of clinical research with support staff and administration Roles focused on contracting, legal, budget negotiation, regulatory/IRB, training, data entry, and outreach
Succession planning	A succession planning strategy to ensure resourcing gaps and needs are continuously met	Career ladder with clear upward mobility for all staff roles Training program for early career, inexperienced staff – includes internships/Co-Ops for coordinator role Succession planning strategy exist
Staff diversity	Representativeness of the PI, staff, and administrators	PI, staff, and administrators represent the population(s) they are serving
Depth of experience	Experience managing the operational components of a research site	Team of cross-functional roles with at least one member in each function that has >3 years’ experience

ACRP = Association of Clinical Research Professionals; IRB = independent review board; PI = principal investigator; SCRS = Society for Clinical Research Sites.

Clinical trial activity cannot take place without a roster of studies and potential research pipeline, so we consider all activities relating to this pursuit under the “trial funnel.” A trial funnel that is predictable and dependable should be built through sponsor connections and partnerships. Metrics for success should relate to communication, fellowship programs, community engagement and professional networks, diversity of the research portfolio, and recruitment methodology (Table 3).

**Table 3.** Measures/predictors of site success: trial funnel

	Metrics for success	Goal(s), site has:
Communication	Proactive sponsor/CRO and patient engagement	Regular, strategic meetings with sponsors/CROs to review pipeline potential Patient-facing approach to education on the importance of clinical trials Staff and patient education and awareness activities on disease state Establish a Communication Plan to identify key stakeholders at the site level
Fellowships	Fellowships in clinical trials offered at the site	Fully developed fellowship program with potential research pipeline Established physician fellowship network to promote and support industry trials
Community engagement network	An existing network to support community engagement	Patient navigators and community engagement staff Defined community engagement strategy with regular meetings, digital footprint, and metrics for success
Professional networks	Engagement with external leaders on industry-sponsored clinical trials	Contracts consulting for pharma Members of societies, collaborations or research groups Published research on industry-sponsored studies
Diversity of research portfolio	Awareness of physician research interest and capacity	Experience in conducting clinical research (broken down by therapy area) Phase I–III trial volume High volume of patients Well-developed model to manage research pipeline
Recruitment methodology	Well-designed recruitment methodology and systems to support capacity to pre-screen	Access to patient insights and local population health Transportation provided Mobile vans in communities Proactive engagement of patients on clinical trials History of meeting enrollment goals Growing omnichannel recruitment methodology Patient-centric consent documentation process

CRO = contract research organization.

## Infrastructure

### Administrative factors

As clinical trials grow in complexity, administrative tasks, such as trial initiation, regulatory compliance, vendor oversight, and workflow management, become increasingly burdensome for study sites [3]. Challenges include procuring and integrating clinical trial management systems (CTMS), streamlining access to medical records, coordinating staff resourcing, and ensuring adherence to protocol-specific requirements. In addition, budgeting these tasks can be challenging, especially for research-naïve sites. The growing number of vendors and the need for specialized training further compound these difficulties.

To alleviate these barriers, sponsors and sites can take several steps. At the outset, it is critical for sponsors and investigators to engage with site administrative leadership. Fostering better communication with administrative staff is important to convey the benefit to sites of participation in trials and to more effectively implement the mitigative actions.

Investing in CTMS technology can improve overall efficiency of trial management by streamlining research operations. For example, CTMS can be used to track trial timelines and site performance and to streamline financial management. A central system such as CTMS is particularly beneficial with the emergence of decentralized trials [12].

Improving the quality and accessibility of site data would enable tailored support for sites. A standardized feasibility assessment across trials and sponsors could reduce operational redundancies. For all new technologies used, training will need to be provided to ensure successful integration.

Site success can be tracked using metrics relating to appropriate facilities, equipment, processes training plans, technology, and the financial health required to support trial activity (Table 1).

### Site financial factors

There are financial challenges throughout the lifecycle of a clinical program, from initial study budget negotiation processes through to patient engagement and payments [13]. Inexperience in conducting studies can make it harder to negotiate a sufficient budget to run trials and generate revenue for long-term sustainability. This can be due to underestimating the full cost of running a clinical trial. A more collaborative approach to the partnership between sponsor and site should encourage shared responsibility to ensure the budget covers all study-related costs, as this will result in a successful trial and is mutually beneficial. To streamline budget negotiations and study startup and reduce administrative burden, technology solutions to automatically populate contracted rates into payment tools and to automate invoicing, are recommended [13].

Enhancing support for currently underfunded activities, such as screen failures, training, startup costs, recruitment, pre-screening, serious adverse event reporting and vendor management, could also help improve the overall sustainability and effectiveness of study sites.

The financial margins of some sites can be exceptionally narrow, and holdbacks or missed payments from sponsors can lead to a critical shortage of operating capital and can contribute to an inability of sites to meet their payroll and pay bills [13,14]. The financial pressures faced by sites have been compounded by general cost inflation, which has risen sharply in recent years [15]. Sponsors can support sites by eliminating holdbacks on study budgets, and ensuring that all study payments are made on time and monthly [9]. Automated solutions are available to support monthly payments in an accurate, compliant, and timely manner [13]. Upfront financial support can benefit sponsors as much as sites, by empowering sites to streamline and accelerate timelines, potentially leading to earlier data availability [16].

Overall, many of the financial challenges faced by sites could be addressed with appropriate support from study sponsors/CROs, including more long-term strategic thinking. Improving efficiencies in administrative tasks becomes easier as the number of trials conducted increases, which in turn results in greater profit and sustainability for future trials.

### Technological factors

Sponsors and CROs are adopting new technologies to help streamline and accelerate the implementation of increasingly complex trials, with many sites embracing Cloud-based solutions [17]. Clinical technology has been evolving at a rapid pace and some 60% of sites are now using ≥20 technology systems on a daily basis [18].

The adoption of new technology is time-consuming and can become burdensome due to the variety of systems used across studies and the short timelines available for learning new tools. As sites are generally not made aware of the technology required for a specific study during its feasibility and early planning process, they are unable to make educated decisions about their technology capacity for participation [18]. Furthermore, some technology infrastructure, such as a comprehensive electronic medical record (EMR) solution, can be an added expense that sites may not be able to afford.

Sites want to use technology but need simpler ways to engage with digital tools. Multiple standalone systems, each with its own portal and sign-in page, can lead to fatigue, limited data flow between systems and information being entered multiple times [19,20]. In addition, each study and sponsor may have different technological tools, so if the site is trying to scale up, they will likely be required to use a range of different systems.

Sponsors and associated CROs could help to reduce the technology burden of their sites through both their choice of technologies, and their approach to communication and training. Adopting technologies that enable cost-effective trial management, such as Cloud-based systems [19], is likely to be preferable to those requiring local on-site data handling or excessive user input. A long-term programmatic approach considers beyond the return on investment of the immediate trial and rather to a broader scope of multiple trials and multiple sponsors. This approach is likely to lead to investment in technologies that sites can keep for use across future trials.

It would help sites greatly to be informed about the details of technologies required for a forthcoming clinical study as early as possible in the consultation process [3]. Sponsors/CROs should then support their sites by providing the necessary training on any new platforms to be used [3]. This involves discussing and planning when training occurs, how training occurs, and the workforce challenges that lead to the need for re-training. There may also be scope for working with sites to assess how their current technology can be utilized more effectively, for example to identify, enroll, and consent potential study participants [3].

## Workforce

### Workforce factors

Staffing is a key challenge faced by study sites [3], largely due to a shortage of experienced clinical research coordinators (CRCs) [1]. There is a paucity of new entrants into the profession [21] as well as issues with staff retention. The turnover rate of patient-facing research personnel increased from 10–37% pre-COVID-19 to 35–61% in 2023 [15]. Reasons include changes in workforce expectations, competitive offers from other organizations, and burnout due to difficulty with patient recruitment, administrative burden, and training/technology overload [15]. A lack of centralized training programs is another factor affecting turnover of CRC staff [22].

The scarcity of resourcing also affects roles specific to budget and contract analysis, and regulatory submissions, creating additional delays in study startup timelines [3]. Challenges posed by workforce issues have a direct impact on the success of clinical trials. Motivating administrative staff can be a challenge due to the perceived financial burden of the trial, as they may struggle to understand the value narrative and profit potential for conducting industry-sponsored trials at their site [2]. These challenges lead to high staff turnover and recruitment difficulties, resulting in a lack of continuity, delays and inconsistencies in data collection and quality, difficulty maintaining accurate records and tracking study progress, an increased risk of compliance issues and subsequent penalties and delays, missed enrollment goals, longer onboarding with increased training and oversight costs, and loss of revenue due to curtailed patient enrollment [15].

It is important to address these workforce challenges through sponsor commitment to support financially with resources, and open communication regarding the economic value of conducting the trial at a local site. In fostering relations between sponsors and sites in this way, the interaction may be viewed by both parties as a balanced, mutually beneficial collaboration. It is also important to be upfront with administrators. Developing this partnership with a long-term view will instill commitment and encourage transparent dialog regarding site challenges without fear of loss of business. This will allow site-specific recommendations to be developed [15].

All parties at both sponsor and site level may benefit from jointly developing standardized, competency-based definitions of required professional roles in clinical research to help evaluate future applicants, better enable the professional growth of individuals throughout their careers, and increase their recognition and value in interprofessional collaborations [21]. Sites may also provide additional training and growth opportunities for their staff, including training for new physicians on how to manage the business aspects of clinical trial management [3], critical for long-term site sustainability and success.

A central team concerned with clinical research conduct should incentivize all those involved through a transparent career development pathway. Metrics for success should relate to administrative leadership engagement, operational mentorship, and investigator engagement. In addition, the metrics should assess the presence of models for sourcing and training junior investigators, succession planning, and depth of experience, and for ensuring that resource and staff diversity are aligned to trial requirements (Tables 2 and 3).

## Trial funnel

### Trial funnel factors

Long-term sustainability and appropriate scale of sites requires a steady flow of clinical trials. Infrastructure and workforce challenges affect a site’s ability to open new clinical trials, which can have significant implications for the entire industry, including delays in drug development, reduced patient access to clinical trials, and increased costs [3]. It is important for sites to have a trial funnel, by which we mean a sufficient volume of new clinical trials, to provide financial security, optimize sponsor relationships, and streamline processes. Similar to business scale up, scale up of trial sites would depend on the demand and loyalty of customers (sponsors).

As discussed above, clinical trial sites commonly face financial challenges [13]. A single study typically does not cover the costs of the necessary personnel to manage clinical trials. Therefore, if a site only conducts a few clinical trials at a time, they often operate while in a financial deficit or without sufficient and appropriate resources. As a result, once the project ends sites may have to terminate employee contracts of those that were hired solely for the study, contributing to a high workforce turnover rate [22]. A consistent trial funnel can enable a site to properly resource their programs and scale their business [1].

Additionally, when a clinical trial funnel is in place sponsors are more likely to trust a site’s ability to enroll patients and provide clean data, which helps to ensure that sponsors will continuously select that site for upcoming studies. A stable base of sponsors positively impacts a site’s financial planning and eases administrative burden by allowing for efficiencies such as expedited feasibility, pre-negotiated contracting language and rates, and programmatic resourcing. The ability to meet sponsor demands and establish a reputation as a preferred site for clinical programs is an external driver for site sustainability [23].

When sites have a strong trial pipeline, they have the capacity to develop long-term solutions to streamline processes. Operational models that are structured to scale with efficiency lead to long-term sustainability [24]. Assessing a site’s current capacity and identifying opportunities to develop their trial funnel can increase financial stability, sponsor placement of clinical trials, and study execution, which combined improves overall data quality and patient experience [25].

## Conclusions

The current trial-by-trial approach to site partnerships has not kept pace with the increasing complexity of clinical research. As a result, research sites continue to face operational barriers, including workforce shortages and infrastructure limitations, that hinder their ability to sustain clinical trial activity.

To address these challenges, sponsors and their associated CROs must adopt a programmatic approach to site engagement, one that moves beyond short-term, study-specific support to long-term, strategic investment. Establishing collaborative agreements with clear success metrics can provide sites with the stability needed to enhance infrastructure, retain skilled personnel, and build a sustainable trial pipeline. Additionally, improving transparency around clinical trial pipelines and strengthening site relationship management will foster a more efficient and cooperative research ecosystem.

By proactively reshaping sponsor-site partnerships, the industry can drive meaningful change, ensuring research sites are equipped to meet growing clinical trial demands while maintaining high-quality execution and compliance.
